# The Role of Plant Growth Regulators in Modulating Root Architecture and Tolerance to High-Nitrate Stress in Tomato

**DOI:** 10.3389/fpls.2022.864285

**Published:** 2022-04-08

**Authors:** Rongting Ji, Ju Min, Yuan Wang, Herbert J. Kronzucker, Weiming Shi

**Affiliations:** ^1^Nanjing Institute of Environmental Sciences, Ministry of Ecology and Environment of the People’s Republic of China, Nanjing, China; ^2^State Key Laboratory of Soil and Sustainable Agriculture, Institute of Soil Science, Chinese Academy of Sciences, Nanjing, China; ^3^Faculty of Land and Food Systems, University of British Columbia, Vancouver, BC, Canada; ^4^School of BioSciences, The University of Melbourne, Parkville, VIC, Australia

**Keywords:** tomato, high-nitrate stress, plant growth regulator, tolerance, root system architecture

## Abstract

Plant growth regulators are known to exert strong influences on plant performance under abiotic stress, including exposure to high nitrate, as occurs commonly in intensive vegetable production. However, direct comparative evaluations of growth regulators under otherwise identical conditions in major crop species are scarce. In this study, tomato (*Solanum lycopersicum* L.) was used as a model crop, and the roles of four common exogenously applied plant growth regulators (MT, melatonin; SA, salicylic acid; HA, humic acid; SNP, sodium nitroprusside) in regulating crop growth were studied under high-nitrate stress. We provide a particular focus on root system architecture and root physiological responses. Our data show that all four growth regulators improve tomato tolerance under high nitrate, but that this occurs to differing extents and via differing mechanisms. Optimal concentrations of MT, SA, HA, and SNP were 50 μmol L^–1^, 25 μmol L^–1^, 25 mg L^–1^, and 50 μmol L^–1^, respectively. MT and SNP produced the strongest effects. MT enhanced root growth while SNP enhanced above-ground growth. Growth of coarse and thin lateral roots was significantly improved. Furthermore, an enhancement of root vitality and metabolism, improved integrity of root cell membranes, and an increase in antioxidant enzyme activities were found, but regulatory mechanisms were different for each growth regulator. Our results show that in particular the application of MT and SNP can improve growth of tomato in intensive vegetable production under high-nitrate stress and that root growth stimulation is of special importance in procuring these beneficial effects.

## Introduction

A high application rate of fertilizer, especially of nitrogen (N) fertilizer is common practice in crop production systems to obtain maximum yield (Ju et al., 2007; Coskun et al., 2017; Min et al., 2021b), including in greenhouse-based vegetable production systems (Shi et al., 2009; Li et al., 2017; Min et al., 2021a). However, N fertilizer use both in field and greenhouse settings is highly inefficient, and often only 10–20% of the N applied in the field can be absorbed by the crop, and consequently, a substantial amount of N remains in soil, is leached, or is lost to the atmosphere (Coskun et al., 2017). Due to the lack of leaching associated with natural rainfall in the greenhouse, and high surface transpiration in long-term intensive planting systems, often leads to serious secondary salinization of the vegetable soil (Darwish et al., 2005; Shi et al., 2009; Min et al., 2011; Wang et al., 2019). Under such conditions, soil NO_3_^–^ content can exceed 1,300 kg ha^–1^ and can account for 67–76% of all anions in the vegetable soil (Zhang et al., 2017; Min and Shi, 2018). Increases in soil NO_3_^–^ ion contents foster the exchange of Ca^2+^ and K^+^ ions from the solid soil phases to form various nitrates, which can result in oxidative damage and metabolic disorders in plants, reduce crop yield, and negatively affect nitrogen metabolism (Wilson and Skeffington, 1994; Du et al., 2017).

Tomato (*Solanum lycopersicum* L.) as a globally utilized fruit vegetable and is mainly cultivated in greenhouses (Jo and Shin, 2021). In 2019, the total production of tomato in the world was 181 million tons, with China accounting for 35% of the total, followed by India and Turkey (FAO, 2020). High nitrate levels in greenhouse soil can, however, produce considerable toxicity conditions for tomato, manifesting both above- and belowground (Cao and Tibbtts, 1998; Wang et al., 2007; Xu et al., 2012). Liang Y. L. et al. (2018) have reported reductions of the fresh weight of shoot and root by 39.02 and 35.42%, respectively (Liang Y. L. et al., 2018). Furthermore, Guo et al. (2018) found that root length was decreased by 15.0, 34.7, and 58.2% following high-nitrate stress for 1, 3, and 5 days, respectively (Guo et al., 2018). Root system architecture, including root length, branching, diameter, and surface area, is critical to soil stability, water and nutrient uptake, and stresses tolerance more generally (González-Hernández et al., 2020). The root morphological changes under high-nitrate stress, however, have not been studied in detail in tomato or other vegetable crops.

Exogenous application of growth regulators can stimulate plant growth, enhance antioxidant capacity, and improve plant tolerance to various abiotic stresses, and selective application of such plant growth regulators can be an effective measure to overcome high-nitrate stress (Liang W. J. et al., 2018; Khan et al., 2021; Xu et al., 2022). Du et al. (2017) showed in cucumber that the exogenous application of growth regulators can lead to growth recovery under high-nitrate stress, and that this is associated with stimulated activities of enzymes involved in N metabolism and with improved carbon assimilation (Du et al., 2017). Several studies have shown that, under high nitrate, the exogenous application of melatonin, salicylic acid, sodium nitroprusside, humic acid, γ-aminobutyric acid, nitradine, 2,4-epibrassinolide, and several other substances can promote plant growth and improve stress tolerance (Wilson et al., 2008; Wani et al., 2017; Hatami et al., 2018; Chen et al., 2021). Among these plant regulators, melatonin (MT), salicylic acid (SA), sodium nitroprusside (SNP), and humic acid (HA) were the most commonly used, with excellent potential for improving abiotic stress tolerance in practice. However, direct comparative studies on the relative efficacies and on plant performance of different exogenously applied growth regulators in tomato under high-nitrate stress under otherwise identical conditions have, thus far, been lacking. It has also remained unclear whether the regulatory mechanisms of various kinds of growth regulators are different from one another.

The objectives of this study were: (i) to compare nitrate stress tolerance in tomato under the influence of four common growth regulators (MT, SA, HA, SNP); (ii) to characterize the root morphology changes of tomato following growth regulator application under high-nitrate stress; (iii) to clarify whether the regulatory mechanisms of the growth regulators are shared or differ from one another. It is hoped that our study will provide new insight into the mechanisms of growth regulator action in tomato and provide some novel guidance for the improvement of vegetable cultivation systems.

## Materials and Methods

### Plant Material, Culture Conditions, and Stress Treatment

Seeds of tomato (*Solanum lycopersicum* L. cv. Hezuo 903) were surface-sterilized with 6% H_2_O_2_ (30 min) and germinated on moistened filter in the dark (3 days, 28^°^C), and then transferred and grown hydroponically, using modified Hoagland solution [KNO_3_, 1.0 mmol L^–1^; Ca(NO_3_)_2_, 2.0 mmol L^–1^; KH_2_PO_4_, 200 μmol L^–1^; MgSO_4_, 0.4 mmol L^–1^; Fe-EDTA, 0.1 mmol L^–1^; H_3_BO_3_, 3.0 μmol L^–1^; MnCl_2_, 3.0 μmol L^–1^; CuSO_4_, 0.5 μmol L^–1^; ZnSO_4_, 1.0 μmol L^–1^; (NH_4_)_2_MoO_4_, 0.1 μmol L^–1^; pH 5.8] for 7 days. The experiment was carried out in a growth camber with a 14-h daily light period (200 μmol m^–2^ s^–1^, 28°C) and a 10-h dark period (25°C) (Zhang et al., 2018). Subsequently, 7-day old seedlings were transferred into the high-nitrate solution (NO_3_^–^ concentration, 100 mmol L^–1^) with equal addition of KNO_3_ and Ca(NO_3_)_2_ for excess nitrate stress treatment (Xu et al., 2012; Du et al., 2017; Chen et al., 2021). A NO_3_^–^ ion concentration of 4.5 mmol L^–1^ was used as a control. After 1-, 4-, and 10-day treatments, plants were separated into leaves, stems, and roots, and then fresh weight (FW) and dry weight (DW) were determined.

Four common regulators (MT, melatonin; SA, salicylic acid; HA, humic acid; SNP, sodium nitroprusside) were selected in this experiment. The 7-day old seedlings were divided into three groups, namely control (SC), high-nitrate stress (SN), high-nitrate stress + regulators (namely by regulator name + each concentration level). Four general concentrations of each regulator were used (Table 1), and the nitrate stress concentrations treatments were conducted as mentioned above. The nutrient media was changed every 48 h, and the treated seedlings were harvested for analysis after exposure to nitrate treatments for 10 days. All treatments were repeated at least three times.

**TABLE 1:** Concentrations of four exogenous growth regulators (MT, melatonin; SA, salicylic acid; HA, humic acid; SNP, sodium nitroprusside) used in the experiment.

Treatment	MT	SA	HA	SNP
1	25 μmol L^–1^	25 μmol L^–1^	25 mg L^–1^	25 μmol L^–1^
2	50 μmol L^–1^	50 μmol L^–1^	50 mg L^–1^	50 μmol L^–1^
3	100 μmol L^–1^	100 μmol L^–1^	100 mg L^–1^	100 μmol L^–1^
4	200 μmol L^–1^	200 μmol L^–1^	200 mg L^–1^	200 μmol L^–1^

### Analysis of Plant Vegetative Growth and Root Characteristics

The harvested seedlings were used to determine aboveground growth indicators, fresh samples of plants were used to measure plant height, leaf length, leaf width and other indicators with a ruler, and stem thickness was determined with the vernier caliper. Then, root scanning was performed to determine morphological parameters of root growth, including total root length, root surface area, root volume, root tips, and root diameter, using a WinRhizo-LA1600 (Regent Instruments Inc., Quebec, QC, Canada) root analysis instrument (Ji et al., 2017).

### Measurement of Root Vitality, Electrolyte Leakage, Lipid Peroxidation, and Antioxidant Enzymes

Determination of root vitality (Chang et al., 2012): Fresh terminal roots were incubated for 2 h at 37°C in darkness with a solution mixture of 0.4% 2,3,5-triphenyltetrazolium chloride and 0.1 mol L^–1^ phosphate buffer, and then 2 mL, 1 mol L^–1^ sulfuric acid was added to stop the reaction. Equal amounts of fresh roots from the same plants were mixed with 2 mL, 1 mol L^–1^ sulfuric acid, which was the comparison treatment. Then, the treated roots were macerated with ethyl acetate and quartz sand for extracting the red-colored formazan (80^°^C, 15 min) that was then quantified spectrophotometrically at 485 nm.

Determination of electrolyte leakage (EL) (Endeshaw et al., 2015): Fresh roots were incubated at 32°C for 2 h in test tubes, which contained 10 mL of double-distilled water for determining initial electrical conductivity (EC1). After heating treatment in boiling water for 20 min, a second electrical conductivity (EC2) measurement was taken. Simultaneously, the electrical conductivity of background distilled water was determined as the third electrical conductivity (EC3). Then, EL was calculated as (EC1-EC3)/(EC2-EC3) × 100%.

Determination of total soluble protein (Song et al., 2015): Fresh roots were mixed with ice-chilled phosphate buffer (pH 7.8) that contained 0.1 mmol L^–1^ EDTA. Then, the resulting homogenate was centrifuged at 4,000 rpm for 20 min, and the supernatant was collected for determination of protein at 595 nm (using BSA solution as standard).

Determination of malondialdehyde (MDA) (Aziz et al., 2018): Fresh roots were ground with 0.1% trichloroacetic acid, and the mixture was centrifuged at 10,000 rpm for 5 min. 1 mL of protein-free supernatant was mixed with 0.25 mL 0.5% thiobarbituric acid, heated with boiling water for 20 min, placed on ice to stop the reaction, and then absorbance at 450, 532, and 600 nm was determined.

Determination of antioxidant enzymes (Aziz et al., 2018): Fresh roots were homogenized with phosphate buffer (pH 7.8) that contained 0.2 mmol L^–1^ EDTA and 2% insoluble polyvinylpyrrolidone, with a chilled pestle and mortar. The homogenate was centrifuged at 12,000 rpm for 20 min, and the supernatant was used for determining enzyme activities. SOD activity was assayed by measuring 50% inhibition of the photochemical reduction per unit time of nitro-blue tetrazolium. CAT activity was measured by UV spectrophotometer, and then calculated as micromoles of H_2_O_2_ oxidized per minute per milligram of total soluble protein.

### Statistical Analysis

The results were statistically analyzed by analysis of variance (ANOVA) and a Duncan multiple-range comparison test with SPSS (SPSS Inc., Chicago, IL, United States). Difference alphabets among treatments indicate statistical significance at the *P* < 0.05 level, and the data are represented as the means ± SE. All graphs were generated using Origin 8.5 (OriginLab Corporation, Northampton, MA, United States). Principal component analysis (PCA) was conducted to analyze and visualize the effect of regulators types and concentration levels on related growth characteristics using CANOCO (Version 4.5, Plant research international, Wageningen, Netherlands).

## Results

### Root Biomass of Tomato Responds Strongly Under High-Nitrate Stress

**FIGURE 1:**
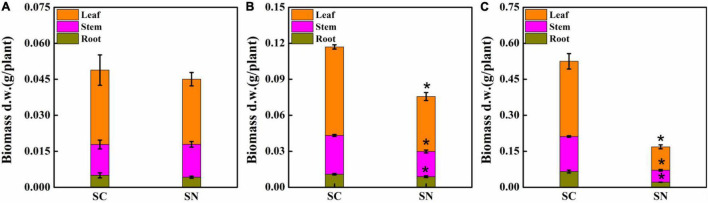
Effects of high-nitrate stress on tomato biomass of roots, stems, and leaves after 1-day **(A)**, 4-day **(B)**, and 10-day **(C)** treatment. SC, control; SN, high-nitrate stress treatment. Asterisks indicate significant differences between SC and SN treatments at the *P* < 0.05 level.

Under high-nitrate stress, the growth of tomato plants was significantly inhibited in terms of roots, stems, and leaves after varying periods of exposure (Figure 1). After 1-day treatment, a 16.00% reduction of root weight was observed compared to the control treatment, and the inhibition rate of leaf growth was observed at 12.35%, while stem growth was not inhibited. After 4 days, the biomass of root, stem, and leaf was decreased by 18.75, 35.15, and 37.84%, respectively. After 10 days, more significant differences were observed: biomass of root, stem, and leaf were 67.44, 65.99, and 68.83% lower for plants under nitrate stress treatment, respectively. These data show that the root is the most sensitive tissue and is inhibited first under high-nitrate stress. Clearly, maintaining proper root growth and function is essential for sustainable growth of tomato plants under high-nitrate stress.

**FIGURE 2:**
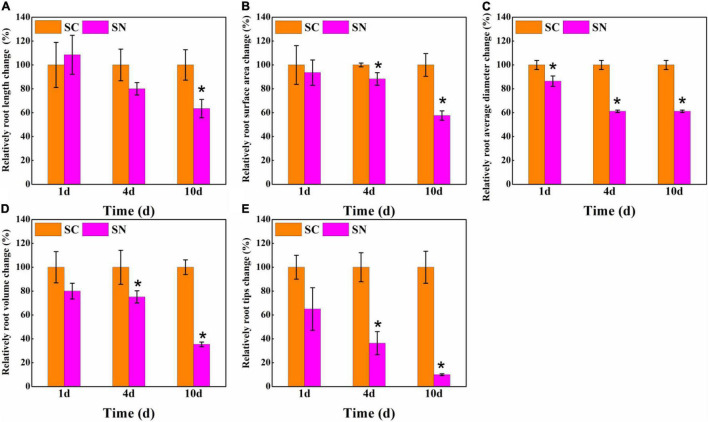
Effects of high-nitrate stress on relative root architecture change of tomato after 1-day, 4-day, and 10-day treatment. **(A)** Total root length; **(B)** Root surface area; **(C)** Root diameter; **(D)** Root volume; **(E)** Root tips. SC, control; SN, high-nitrate stress treatment. Asterisks indicate significant differences between SC and SN treatment at the *P* < 0.05 level.

Furthermore, the root morphology characteristics were analyzed under both control and high-nitrate treatment. As shown in Figure 2, the total root length, root surface area, root diameter, root volume, and root tip number were changed by 108.52, 93.56, 86.41, 80.00, and 65.00% after 1 day under high-nitrate treatment, respectively. After 4 days, considerable growth inhibition was found for tomato roots. A 20.02, 11.73, 38.82, 24.91, and 63.64% reduction was observed for total root length, root surface area, root diameter, root volume, and root tip number, respectively. After 10 days, total root length, root surface area, root diameter, root volume, and root tip number were reduced to 63.44, 57.59, 61.18, 35.40, and 10.00% compared to the control treatment, respectively. Among these indicators, the root tips were most inhibited under nitrate-stress conditions, while total root length was less inhibited, and the order of the degree of inhibition for root indicators was: root tips number > root volume > root diameter > root surface area > total root length.

### Morphological Parameters Show Different Performances After Exposure to Different Plant Growth Regulators

**FIGURE 3:**
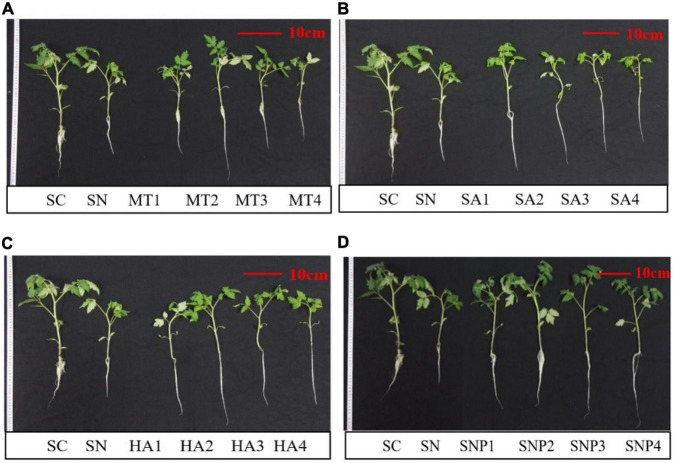
Effects of different concentrations of **(A)** MT, **(B)** SA, **(C)** HA, and **(D)** SNP on tomato growth under high-nitrate stress. SC, control; SN, high-nitrate stress treatment; MT, melatonin; SA, salicylic acid; HA, humic acid; SNP, sodium nitroprusside; MT1–4 represented SN + 25 μmol L^–1^, 50 μmol L^–1^, 100 μmol L^–1^, 200 μmol L^–1^ MT; SA1–4 represented SN + 25 μmol L^–1^, 50 μmol L^–1^, 100 μmol L^–1^, 200 μmol L^–1^ SA; HA1–4 represented SN + 25 mg L^–1^, 50 mg L^–1^, 100 mg L^–1^, 200 mg L^–1^ HA; SNP1–4 represented SN + 25 μmol L^–1^, 50 μmol L^–1^, 100 μmol L^–1^, 200 μmol L^–1^ SNP. To allow for direct comparison, the same plants for SC and SN were used in the four panels.

**FIGURE 4:**
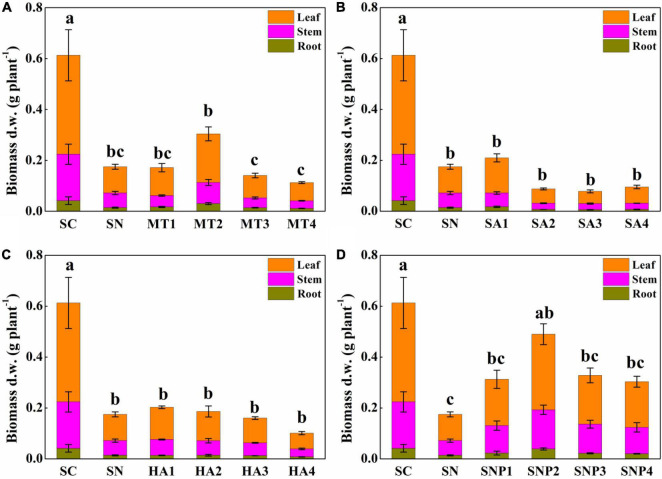
Effects of different concentrations of **(A)** MT, **(B)** SA, **(C)** HA, and **(D)** SNP on root, stem, and leaf biomass of tomato under high-nitrate stress. SC, control; SN, high-nitrate stress treatment; MT, melatonin; SA, salicylic acid; HA, humic acid; SNP, sodium nitroprusside; MT1–4 represented SN + 25 μmol L^–1^, 50 μmol L^–1^, 100 μmol L^–1^, 200 μmol L^–1^ MT; SA1–4 represented SN + 25 μmol L^–1^, 50 μmol L^–1^, 100 μmol L^–1^, 200 μmol L^–1^ SA; HA1–4 represented SN + 25 mg L^–1^, 50 mg L^–1^, 100 mg L^–1^, 200 mg L^–1^ HA; SNP1–4 represented SN + 25 μmol L^–1^, 50 μmol L^–1^, 100 μmol L^–1^, 200 μmol L^–1^ SNP. Different letters indicate significant statistical differences at the *P* < 0.05 level as determined by Duncan’s multiple range test.

After adding plant growth regulators, the degree to which plant growth was alleviated was different for each regulator, and there existed a certain dose effect (Figure 3). After applying MT, root, stem, and leaf biomass were changed by −20.71–114.5, −48.00–43.49, −30.99–85.18%, respectively, compared to stressed seedlings in the absence of growth regulators. It was particularly noteworthy that the MT2 (50 μmol L^–1^) treatment had a significant effect on offsetting the decline in plant growth. When adding SA, the growth inhibition was not considerably alleviated. On the contrary, a high concentration of SA (≥50 μmol L^–1^) even had a partial inhibitory effect on plant growth: the growth of root, stem, and leaf of tomato plants after adding SA was changed by −57.62–23.81, −58.29–5.78, −53.52–33.67%, respectively. The application of HA promoted root, stem, and leaf biomass by −44.29–1.19, −45.75–7.69, −39.96–22.69%, respectively. The treatment with HA1 (25 mg L^–1^) had a significant positive, protective effect on plant growth, and the effect of HA on aboveground tissues was stronger than on the root system. The biomass of root, stem, and leaf was enhanced by 44.05–178.6, 80.11–167.27, 73.50–187.5%, respectively, after application of SNP; especially SNP2 (50 μmol L^–1^) had a strong effect on protecting plant growth (Figure 4). In conclusion, various plant growth regulators showed different effects on alleviating high-nitrate stress, and MT and SNP possessed the strongest alleviatory effects among the four regulators.

To understand the effects of plant growth regulators on plant growth indices, plant height, stem diameter, number of leaves, leaf length, leaf width, and leaf area of tomato were investigated. After using plant growth regulators, the plant height was promoted by 15.67–20.00, 13.50–18.17, 14.67–19.33, and 18.17–25.67% for the application of MT, SA, HA, and SNP, respectively. The stem diameter was less promoted, and the indicator was changed by −13.00–17.00, −31.00–2.00, −14.00–8.00, and 0–22.00% after using MT, SA, HA and SNP, respectively. When considering leaf growth, leaf number, leaf length, leaf width, and leaf area were changed by −36.84–43.86, −18.30–17.65, −15.66–27.71, and −27.90–50.35% after providing plant growth regulators, respectively (Table 2). The promoting effect for the morphological parameters was ordered as follows: SNP > MT > HA > SA. The optimal concentrations of MT, SA, HA, and SNP under high-nitrate stress were 50 μmol L^–1^, 25 μmol L^–1^, 25 mg L^–1^, and 50 μmol L^–1^, respectively.

**TABLE 2:** Effects of exogenously applied growth regulators on morphological parameters of tomato under high-nitrate stress.

Treatment	Height (cm)	Stem diameter (mm)	Number of leaves	Leaf length (cm)	Leaf width (cm)	Leaf area (cm^2^)
SC	28.67 ± 0.88a	4.77 ± 0.15a	27.67 ± 1.86a	7.77 ± 0.65a	3.83 ± 0.38a	22.69 ± 4.11a
SN	18.17 ± 0.44efg	3.33 ± 0.12de	19.00 ± 1.00bcdef	5.10 ± 0.21bcdef	2.77 ± 0.15cdef	10.58 ± 0.64cd
MT1	17.33 ± 0.33fgh	2.93 ± 0.12fg	18.00 ± 1.15cdefg	4.63 ± 0.38defg	2.67 ± 0.067*cdef*	9.30 ± 0.98*cd*
MT2	20.00 ± 1.15de	3.90 ± 0.058bc	22.33 ± 1.20b	5.57 ± 0.23bc	2.43 ± 0.12ef	10.20 ± 0.90cd
MT3	16.17 ± 0.17ghi	3.03 ± 0.033efg	16.67 ± 0.88*efgh*	4.67 ± 0.33cdefg	2.67 ± 0.17cdef	9.38 ± 1.08cd
MT4	15.67 ± 0.60hij	2.90 ± 0.10g	15.33 ± 0.33fghi	4.43 ± 0.067efg	2.37 ± 0.067ef	7.87 ± 0.30d
SA1	18.16 ± 0.44efg	3.40 ± 0.17d	18.67 ± 1.45bcdef	5.57 ± 0.067bc	3.07 ± 0.30bcd	12.83 ± 1.36bc
SA2	14.67 ± 0.33ij	2.73 ± 0.12g	13.00 ± 1.15i	4.47 ± 0.14efg	2.37 ± 0.067ef	7.94 ± 0.46d
SA3	13.50 ± 0.29j	2.30 ± 0.10h	12.00 ± 1.73hi	4.30 ± 0.25fg	2.50 ± 0ef	8.06 ± 0.47d
SA4	14.33 ± 0.44ij	2.73 ± 0.033g	15.00 ± 1.00fghi	4.17 ± 0.17g	2.43 ± 0.12ef	7.63 ± 0.63d
HA1	19.33 ± 0.33def	3.60 ± 0.10cd	19.00 ± 1.15bcdef	5.43 ± 0.067bcd	2.93 ± 0.067cde	11.95 ± 0.24bcd
HA2	19.17 ± 0.17def	3.53 ± 0.18d	18.67 ± 1.86defg	5.10 ± 0.10bcdef	2.57 ± 0.033def	9.82 ± 0.28cd
HA3	17.33 ± 0.33fgh	3.27 ± 0.12def	17.00 ± 1.00defg	4.83 ± 0.17cdefg	2.33 ± 0.17f	8.44 ± 0.54d
HA4	14.67 ± 0.33ij	2.87 ± 0.13g	14.33 ± 0.88ghi	4.50 ± 0.29efg	2.50 ± 0.00ef	8.44 ± 0.54d
SNP1	23.16 ± 1.48c	3.40 ± 0.15d	22.00 ± 1.53bc	5.43 ± 0.30bcd	3.17 ± 0.17bc	12.98 ± 1.40bc
SNP2	25.67 ± 0.67b	4.07 ± 0.033b	27.33 ± 1.20a	6.00 ± 0.00b	3.53 ± 0.27ab	15.90 ± 1.20b
SNP3	23.67 ± 1.33bc	3.47 ± 0.033d	21.00 ± 1.00bcd	4.83 ± 0.33cdefg	2.83 ± 0.17cdef	10.31 ± 1.14cd
SNP4	21.00 ± 1.00d	3.33 ± 0.088de	20.00 ± 0.58bcde	5.27 ± 0.37bcde	2.73 ± 0.15cdef	10.88 ± 1.35cd

*SC, control; SN, high nitrate stress treatment; MT, melatonin; SA, salicylic acid; HA, humic acid; SNP, sodium nitroprusside; MT1–4 represented SN + 25 μmol L^–1^, 50 μmol L^–1^, 100 μmol L^–1^, 200 μmol L^–1^ MT; SA1–4 represented SN + 25 μmol L^–1^, 50 μmol L^–1^, 100 μmol L^–1^, 200 μmol L^–1^ SA; HA1–4 represented SN + 25 mg L^–1^, 50 mg L^–1^, 100 mg L^–1^, 200 mg L^–1^ HA; SNP1–4 represented SN + 25 μmol L^–1^, 50 μmol L^–1^, 100 μmol L^–1^, 200 μmol L^–1^ SNP. Different letters indicate significant statistical differences at the P < 0.05 level as determined by Duncan’s multiple range test.*

### Root Morphology Parameters Show Different Performances After Exposure to Different Plant Growth Regulators

Both nitrate stress and plant growth regulators had a significant effect on the root morphology of tomato, and different performance was observed for different plant growth regulators (Table 3). Total root length was changed by −27.79–79.54, −46.03–18.97, −38.24–15.91, and −4.40–65.70% after application of MT, SA, HA, and SNP, respectively. In particular, application of MT significantly relieved growth suppression brought about by nitrate stress, and root growth under MT2 recovered to pre-stress levels. Root surface area was enlarged by −49.25–137.61%, while the application of SA made no significant difference to this value. The regulators also significantly increased root volume, by −18.59–59.98, −124.26–16.42, −59.51–7.55, and 31.16–68.28%, after application of MT, SA, HA, and SNP, respectively. Moreover, root tip number distinctly recovered after growth regulator treatment, and this number, which indicates the growth of lateral roots, was enhanced by 76.92–433.33, −43.59–74.36, −28.20–102.56, and 66.67–566.67% for MT, SA, HA, and SNP, respectively. Therefore, compared among all plant growth regulators, MT and SNP offset the decline of root growth, especially lateral roots, under high-nitrate stress most markedly.

**TABLE 3:** Effects of exogenously applied plant growth regulators on root morphology of tomato under high-nitrate stress.

Treatment	Total root length (cm)	Root surface area (cm^2^)	Root diameter (mm)	Root volume (cm^3^)	Root tips
SC	855.70 ± 34.48b	64.83 ± 8.04a	0.21 ± 0.011a	0.35 ± 0.048a	112.50 ± 22.77a
SN	557.87 ± 31.63cdef	22.11 ± 2.74cdefg	0.13 ± 0.0043efg	0.076 ± 0.012cdef	13.00 ± 3.51cde
MT1	626.88 ± 25.95cde	28.25 ± 3.68cd	0.14 ± 0.0023def	0.10 ± 0.012cde	35.67 ± 8.84cd
MT2	1001.58 ± 40.50a	48.77 ± 6.22b	0.15 ± 0.0009cd	0.19 ± 0.025b	69.33 ± 3.84b
MT3	547.36 ± 24.87def	25.31 ± 2.64cdef	0.15 ± 0.0043de	0.093 ± 0.012cdef	31.33 ± 2.60cde
MT4	402.82 ± 43.67gh	17.90 ± 1.54cdefg	0.14 ± 0.0077def	0.064 ± 0.0056cdef	23.00 ± 3.61cde
SA1	663.71 ± 6.36b	27.42 ± 1.83cde	0.13 ± 0.0034efg	0.090 ± 0.0048cdef	22.67 ± 2.33cde
SA2	336.18 ± 1.26h	12.67 ± 0.21fg	0.12 ± 0.0019g	0.038 ± 0.0012ef	9.33 ± 1.86cde
SA3	301.07 ± 15.36h	11.22 ± 1.53g	0.12 ± 0.0055g	0.034 ± 0.0063f	7.33 ± 1.33e
SA4	379.43 ± 17.27h	15.08 ± 2.14defg	0.13 ± 0.0048fg	0.048 ± 0.0086def	9.00 ± 1.73de
HA1	549.38 ± 12.03cdef	23.18 ± 1.79cdefg	0.13 ± 0.0029efg	0.078 ± 0.0078cdef	26.33 ± 6.17cde
HA2	646.62 ± 31.29c	25.74 ± 5.58cdef	0.13 ± 0.0031fg	0.082 ± 0.019cdef	19.67 ± 3.18cde
HA3	491.14 ± 31.81fg	20.51 ± 0.77cdefg	0.13 ± 0.0058efg	0.068 ± 0.0035cdef	16.33 ± 1.86cde
HA4	344.50 ± 23.05h	14.30 ± 0.71efg	0.13 ± 0.0024efg	0.047 ± 0.0018def	9.33 ± 3.38cde
SNP1	533.31 ± 34.49def	29.23 ± 6.64c	0.17 ± 0.016bc	0.13 ± 0.042c	33.00 ± 13.5cde
SNP2	924.41 ± 67.43ab	52.54 ± 7.17b	0.18 ± 0.0044b	0.24 ± 0.032b	86.67 ± 8.67b
SNP3	634.98 ± 48.48cde	29.63 ± 4.03c	0.15 ± 0.0010de	0.11 ± 0.013cd	36.00 ± 10.3c
SNP4	631.65 ± 45.20cde	29.52 ± 2.10c	0.15 ± 0.0008de	0.11 ± 0.0074cd	21.67 ± 1.45cde

*SC, control; SN, high nitrate stress treatment; MT, melatonin; SA, salicylic acid; HA, humic acid; SNP, sodium nitroprusside; MT1–4 represented SN + 25 μmol L^–1^, 50 μmol L^–1^, 100 μmol L^–1^, 200 μmol L^–1^ MT; SA1–4 represented SN + 25 μmol L^–1^, 50 μmol L^–1^, 100 μmol L^–1^, 200 μmol L^–1^ SA; HA1–4 represented SN + 25 mg L^–1^, 50 mg L^–1^, 100 mg L^–1^, 200 mg L^–1^ HA; SNP1–4 represented SN + 25 μmol L^–1^, 50 μmol L^–1^, 100 μmol L^–1^, 200 μmol L^–1^ SNP. Different letters indicate significant statistical differences at the P < 0.05 level as determined by Duncan’s multiple range test.*

**FIGURE 5:**
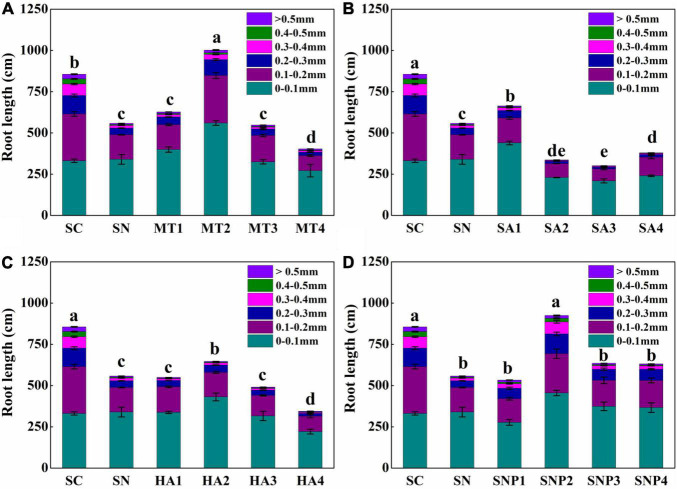
Root-length proportions of sections with different diameters in response to high-nitrate stress of **(A)** MT, **(B)** SA, **(C)** HA, and **(D)** SNP application treatment. SC, control; SN, high-nitrate stress treatment; MT, melatonin; SA, salicylic acid; HA, humic acid; SNP, sodium nitroprusside; MT1–4 represented SN + 25 μmol L^–1^, 50 μmol L^–1^, 100 μmol L^–1^, 200 μmol L^–1^ MT; SA1–4 represented SN + 25 μmol L^–1^, 50 μmol L^–1^, 100 μmol L^–1^, 200 μmol L^–1^ SA; HA1–4 represented SN + 25 mg L^–1^, 50 mg L^–1^, 100 mg L^–1^, 200 mg L^–1^ HA; SNP1–4 represented SN + 25 μmol L^–1^, 50 μmol L^–1^, 100 μmol L^–1^, 200 μmol L^–1^ SNP. Different letters indicate significant statistical differences at the *P* < 0.05 level as determined by Duncan’s multiple range test.

Given the routine assessment of root diameter as an indicator of nutrient uptake capacity and of growth status, the effect of growth regulators on root lengths of varying diameters was further examined (Figure 5). Following nitrate stress, root growth, especially for coarse roots, was significantly inhibited. However, when applying MT, the growth of both fine and coarse roots was strengthened. For example, with the MT2 treatment, the diameter of 0–0.1 mm and >0.1 mm root was increased to 220.22 and 223.48 cm, respectively. SA1, by contrast, significantly enhanced in particular fine root growth. Root lengths of roots with a diameter of 0–0.1 mm under SA1 treatment were increased by 5.30% under nitrate stress, while root lengths of roots with a diameter of 0.2–0.5 mm were decreased by 5.04%. HA influenced coarse roots only slightly, while fine roots were enhanced by almost 100 cm under the HA2 treatment. With SNP treatment, coarse roots were promoted more pronouncedly, and root lengths of roots with a diameter of 0.2–0.3 and 0.3–0.4 mm were enhanced by 5.66 and 5.00%, respectively. When applying SNP2, root lengths of roots with diameters of 0.2–0.3 and 0.3–0.4 mm were increased by 79.02 and 56.91 cm, respectively. Therefore, fine and coarse lateral roots were both promoted under MT treatment, while SNP had a more positive effect on the growth of coarse roots, and SA significantly promoted fine roots with no significant changes observed for HA.

### Root Activity, Electrolyte Leakage, Cell-Metabolic Activity, and Antioxidant Enzyme Activity Exhibit Differential Performance With Provision of Different Plant Growth Regulators

Root activity, electrolyte leakage, cell-metabolic activity, and antioxidant enzyme activity are all key indicators of plant performance under stress, and such is also the case under high-nitrate stress (Table 4). Compared with the SN treatment, the application of MT2 strongly supported root vitality; the value obtained in the tetrazolium reduction vitality test increased 3.82 times, while only an infinitesimal decrease was observed for electrolyte leakage. The application of MT2 furthermore decreased the MDA content by 34.24%, while there was no significant difference in SOD and CAT enzyme activity, indicating that these two enzymes do not play a key role in this process. When applying SA1, the root vitality value was increased by 32.37% and electrolyte leakage was decreased by 14.97%. With MDA, significant decrease was observed, and the content was reduced by 29.55%; the activity of SOD was not enhanced but CAT activity was enhanced by 1.10 U/mg prot. Compared with the high-nitrate treatment, exogenous HA1 had no significant effect on root activity, and an 8.37% reduction in electrolyte leakage was observed. In addition, MDA content was significantly decreased by 6.39 nmol/mg prot. CAT enzyme content was increased 1.08 times while SOD was not increased. For SNP, the root vitality in the SNP2 treatment ranked after the MT2 treatment, and the value was 1.65 times that of the SN treatment, while electrolyte leakage was not decreased with SNP2 treatment. MDA content was decreased by 11.04% compared to SN, and a 149.48% increase in CAT activity was observed, while SOD activity was only enhanced by 39.33 U/mg prot.

**TABLE 4:** Effects of exogenously applied growth regulators on root activity, electrolytic leakage, total soluble protein, malondialdehyde (MDA) content, and antioxidant enzyme activities under high-nitrate stress.

Treatment	Root vitality (ug g^–1^ h^–1^)	Electrolytic leakage (%)	MDA (nmol/mg prot)	SOD (U/mg prot)	CAT (U/mg prot)
SC	518.06 ± 55.39a	14.01 ± 1.18f	7.72 ± 0.39h	348.26 ± 14.41de	1.15 ± 0.13g
SN	38.95 ± 4.94e	61.78 ± 2.47bcd	16.58 ± 0.42abc	451.92 ± 67.06cde	3.86 ± 0.41fg
MT1	197.71 ± 17.65bc	53.74 ± 1.45cde	13.89 ± 0.85bcde	617.18 ± 29.80bc	3.88 ± 0.31fg
MT2	187.71 ± 21.57bc	57.42 ± 1.38bcde	10.90 ± 0.47fg	457.28 ± 53.57bcde	3.85 ± 0.30fg
MT3	165.96 ± 14.19c	55.08 ± 3.68cde	9.47 ± 0.46gh	394.98 ± 43.98de	3.59 ± 0.59fg
MT4	34.27 ± 0.65e	53.47 ± 2.12cde	14.33 ± 0.67bcde	610.60 ± 85.36bc	7.16 ± 1.31cde
SA1	51.56 ± 6.10e	52.53 ± 0.89de	11.68 ± 0.49defg	389.14 ± 51.40de	4.96 ± 0.29ef
SA2	150.30 ± 35.01cd	55.56 ± 4.66cde	11.53 ± 1.19defg	390.27 ± 17.75de	5.75 ± 0.22def
SA3	260.35 ± 86.28b	60.20 ± 2.47bcd	16.25 ± 1.47ab	330.87 ± 45.71e	3.88 ± 0.31fg
SA4	37.54 ± 2.84e	56.07 ± 1.24cde	11.45 ± 0.47efg	509.07 ± 40.37bcd	4.99 ± 0.20ef
HA1	41.99 ± 6.41e	56.61 ± 2.70cde	10.19 ± 0.12fgh	373.78 ± 27.57de	8.04 ± 0.97cd
HA2	40.69 ± 6.09e	53.84 ± 5.21cde	18.46 ± 1.68a	1009.47 ± 98.64a	15.25 ± 3.56a
HA3	42.43 ± 7.73e	51.54 ± 2.81de	12.94 ± 0.65cdef	493.29 ± 60.63bcde	6.60 ± 0.41def
HA4	50.70 ± 15.12e	49.27 ± 1.69e	18.24 ± 1.02a	609.66 ± 61.92bc	12.73 ± 1.57ab
SNP1	75.48 ± 7.53de	73.94 ± 2.51a	16.37 ± 1.26ab	622.38 ± 43.02bc	12.31 ± 0.92b
SNP2	64.17 ± 8.05de	63.55 ± 0.91bc	14.75 ± 1.39bcde	491.25 ± 35.40bcde	9.63 ± 0.99c
SNP3	119.42 ± 14.58cde	67.59 ± 6.98ab	14.68 ± 0.80abcd	624.88 ± 28.61bcde	7.28 ± 0.30cde
SNP4	174.66 ± 26.78bc	63.57 ± 1.55bc	15.80 ± 1.17abc	508.23 ± 27.11bcd	8.58 ± 0.98cd

*SC, control; SN, high nitrate stress treatment; MT, melatonin; SA, salicylic acid; HA, humic acid; SNP, sodium nitroprusside; MT1–4 represented SN + 25 μmol L^–1^, 50 μmol L^–1^, 100 μmol L^–1^, 200 μmol L^–1^ MT; SA1–4 represented SN + 25 μmol L^–1^, 50 μmol L^–1^, 100 μmol L^–1^, 200 μmol L^–1^ SA; HA1–4 represented SN + 25 mg L^–1^, 50 mg L^–1^, 100 mg L^–1^, 200 mg L^–1^ HA; SNP1–4 represented SN + 25 μmol L^–1^, 50 μmol L^–1^, 100 μmol L^–1^, 200 μmol L^–1^ SNP. Different letters indicate significant statistical differences at the P < 0.05 level as determined by Duncan’s multiple range test.*

### Principal Component Analysis Confirms the Differential Effect of Plant Growth Regulators

**FIGURE 6:**
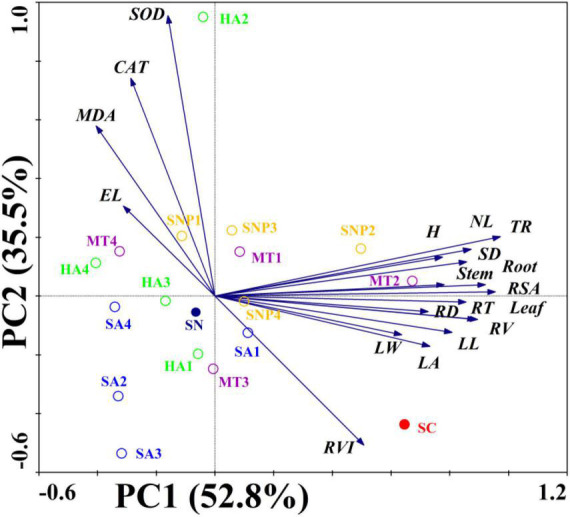
Principal component analysis and factor scores for the selected related growth indicators as the first two principal components. Root, root weight; Stem, stem weight; Leaf, leaf weight; H, plant height; SD, Stem diameter; NL, number of leaves; LL, leaf length; LW, leaf width; LA, leaf area; TRL, total root length; RSA, root surface area; RD, root diameter; RV, root volume; RT, root tips; RVI, root vitality; EL, electrolytic leakage; MDA, MDA content; SOD, SOD enzyme activity; CAT, CAT enzyme activity.

Principal component analysis reveals that the 19 plant growth parameters were divided into PC1 (52.8%) and PC2 (35.5%). Thus, a total of 88.3% of the differences in all indicators could be explained. In addition, these parameters were divided into two categories. For example, root weight, stem weight, leaf weight, plant height, stem diameter, number of leaves, leaf length, leaf width, leaf area, total root length, root surface area, root diameter, root volume, root tips number, and root vitality were distributed in the first and fourth quadrants and showed a positive relationship with nitrate-stress tolerance of tomato. On the other hand, the root, electrolyte leakage, MDA content, SOD and CAT enzyme activity were distributed in the second and third quadrants and showed an opposite relationship with tomato growth parameters (Figure 6). The SNP2 and MT2 treatments had more than 0.50 of the PC1 loadings and were located closer to the SC treatment, suggesting that, under those two treatments, growth of the tomato plant was less stressed and growth indicators could recover to the state prior to the imposition of stress. In addition, a clear separation in PC1 and PC2 loading scores was observed based on the plant growth regulator type and concentration. The SNP treatment was distributed along the PC2 axis, with the PC2 loadings of SNP ranging from 0 to 0.3, while the PC2 loadings of the SA treatment ranged from −0.5 to 0, suggesting a different response and dispersion from the SNP and SA treatments. The PC1 loadings were observed to range from 0 to 0.75 of the MT treatment (except MT4), and the PC1 loadings of HA treatment were located at −0.5–0, suggesting dispersion among the MT and HA treatment. Therefore, the various regulators acted in different ways in the context of tolerance to high-nitrate stress, and the application of SNP2 and MT2 showed the best offset effect of plant growth under the stress condition.

## Discussion

### The Variable Effects of Plant Growth Regulators on Tomato Growth Under High-Nitrate Stress

Inhibited physiological and biochemical growth indicators is a critical phenomenon to environmental stress (Yuan et al., 2015; Du et al., 2017). In this study, the biomass of tomato was significantly inhibited, by 67.44, 65.99, and 68.83% for roots, stems, and leaves under nitrate stress, which is consistent with previous studies in cucumber, spinach, and other vegetables (Xu et al., 2016; Guo et al., 2018). In past studies, the application of plant growth regulators was shown to constitute an effective measure to overcome stresses such as those presented by high nitrate (Du et al., 2018; Liang W. J. et al., 2018; Khan et al., 2021). To date, however, the performances of different regulators under controlled and otherwise identical nitrate-stress conditions have not been evaluated (Zheng et al., 2016; Zhang et al., 2017). In this study, four common plant growth regulators, MT, SA, HA, and SNP were selected to investigate the impacts on plant growth under excess nitrate. The results show that all plant growth regulators affected plant performance when tomato plants were challenged with high levels of nitrate, and changes were observed in a number of key morphological parameters and in root architecture (Figure 4 and Tables 2, 3). MT, in particular, emerged as a positive modulator of root growth and root biomass was enhanced by as much as 114.5%. SNP alleviated nitrate stress by targeting vegetative growth and promoted biomass of stem and leaf by 80.11–167.27% and by 73.50–187.5%, respectively. HA and SA had midler positive effects, and high concentrations of SA could even inhibit tomato growth (Figure 2). As plant growth regulators are substances similar to phytohormones, they can act potently already at low concentrations, while high concentrations can indeed inhibit plant growth (Ljung, 2014; Laplaze et al., 2015). However, the chemical structures of the regulators employed are quite different from one another, and a structure-activity relationships analysis leads to the expectation of differential influences on the processes of growth and differentiation of cells, tissues, and organs (Rademacher, 2015). For instance, melatonin is an amphiphilic tryptophan-derived indoleamine, while SNP acts as an NO donor and can thus directly interfere with ROS-generating and -perpetuating chain reactions (Gupta et al., 2017; Cipolla-Neto and do Amaral, 2018).

### Application of Plant Growth Regulators Accelerates the Development of Coarse and Thin Lateral Roots, Thereby Improving Tomato Tolerance to High-Nitrate Stress

Root architecture is central to the maintenance of nutrient and water acquisition, but is highly sensitive to environmental stresses (Guo et al., 2017). In past studies, high-nitrate stress was linked to severe damage to root growth in tomato, while detailed examinations of root architecture under such conditions have not been undertaken (Guo et al., 2018; Liang Y. L. et al., 2018). In this study, a comprehensive analysis of root morphological changes was carried out. We show that after 10 days of high-nitrate stress, a 67.44% decrease in root biomass (Figure 1) was linked to reductions in total root length, root surface area, root diameter, root volume, and root tip number, which were reduced to 63.44, 57.59, 61.18, 35.40, and 10.00% of control, respectively (Figure 2). Interestingly, root diameters changed considerably under high nitrate as well; a root diameter change by 38.82% was noted overall, in agreement with Zobel et al. (2007), who previously noted changes in fine root diameter in response to increasing nitrate concentrations, with peaks in diameter class observed at 0.17 mm for 1.5 mmol L^–1^ and 0.13 mm for 12 mmol L^–1^ nitrate. As the typical response to nitrate levels, the decrease of root diameter may present a loss in the number of cells and in cell size in the cortical cell layer (Zobel et al., 2007; Zhang and Wang, 2015). After supplying plant growth regulators, root diameters significantly increased, with notable differences between regulators for each root diameter class. The length of each root diameter class integrates the chemical and physical processes associated with root development. MT and SNP improved both fine and coarse root growth, showing their potential in targeting root cell types in each cell file, and consequently, enhancing water and nutrient uptake and effecting nitrate tolerance (Werner et al., 2001; Guo et al., 2017; Wang et al., 2021).

### The Mitigation Mechanisms of Plant Growth Regulators for Root Growth Under High-Nitrate Stress

The nitrate-stress tolerance of tomato depends on a series of complex processes at the physiological and biochemical levels (Ashraf and Harris, 2004). However, whether the mitigation mechanisms of different growth regulators under nitrate stress are shared or are distinct has remained unclear (Du et al., 2018; Guo et al., 2018). In this study, the different plant growth regulators tested showed clearly differential effects. MT has been shown to act as an antioxidant in some studies (Wei et al., 2015). In our study, the MT2 treatment enhanced nitrate tolerance by improving root vitality and decreasing MDA content, while the activities of SOD and CAT enzymes were not influenced, suggesting these two enzymes were not directly involved in scavenging superoxide radicals induced by high-nitrate stress, and other enzymes localized in different subcellular compartments, such as APX, MDHAR, DHAR, GR, and GPX, may contribute to the process. In salt-stressed maize, activities of POD and APX were increased by MT application, while in salt-stressed bermudagrass leaves increased activities in both SOD and POD were observed following MT application (Shi et al., 2015; Jiang et al., 2016). SNP, as NO donor, by contrast, has been shown to markedly improve root growth by inhibiting ethylene production and by promoting antioxidant activity in carnations (Naing et al., 2017). In our study, root vitality and both SOD and CAT activity under SNP2 were enhanced, while MDA content decreased slightly and electrolyte leakage was not decreased, suggesting the antioxidant system contributes to the tolerance mechanism. However, other pathways, in particular phytohormone pathways, such as those influencing ABA/GA balance, should also be further explored (Antonia et al., 2018). In terms of electrolyte leakage, multiple factors, including the activity of ROS-activated outwardly rectifying K^+^ channels, oxidative degradation of the lipid bilayer, and mechanical defects may have contributed to the results in electrolyte leakage to some degree (Mckay and White, 1996; Vadim et al., 2014). The applications of SA1 and HA1 decreased electrolyte leakage and MDA content, coupled to significant increases in CAT content, which is partly consistent with the finding, in Arabidopsis, that SA promotes seed germination under high salinity by modulating antioxidant activity and by reducing H_2_O_2_ content, and is also consistent with HA application contributing to the enzyme synthesis of CAT and POD in almond rootstocks (Hatami et al., 2018; Lee et al., 2010). Clearly, ROS-detoxification systems operate under high-nitrate stress and play a significant role, while the interplay with other factors, such as the activities of key ion transporters and channels, the SOS pathway, calcium homeostasis, phytohormones, transcription factors, mitogen-activated protein kinases, and osmotica such as glycine betaine and proline, should be explored in the future (Tuteja, 2007).

## Conclusion

Exogenous application of plant growth regulators can help in the alleviation of high-nitrate stress. In this study, four common plant growth regulators (MT, SA, HA, SNP) were selected to examine, in the tomato crop model system, whether they share a common mechanism of growth rescue or whether these mechanisms are distinct from one another, with a special emphasis on root growth. Optimal concentrations of MT, SA, HA, and SNP for tomato shoots and roots were 50 μmol L^–1^, 25 μmol L^–1^, 25 mg L^–1^, and 50 μmol L^–1^, respectively. MT and SNP had superior mitigation effects under nitrate stress. After addition of plant growth regulators, the growth suppression of tomato under nitrate challenge was significantly alleviated, especially the growth suppression of lateral roots. In addition, improvements in tomato root vitality and metabolism, the integrity of root cell membranes, and the functioning of the antioxidant system were noted, but these were engaged to differing extents among the regulators examined. Our findings lead to the recommendation to deploy appropriate concentrations of MT and SNP to promote the growth of tomato and other vegetable crops in situations where high-nitrate stress is likely to be encountered, such as in intensive vegetable production systems.

## Data Availability Statement

The original contributions presented in the study are included in the article/supplementary material, further inquiries can be directed to the corresponding author.

## Author Contributions

WS and JM conceived and designed the experiments. RJ performed the experiments. JM and YW analyzed the data. WS contributed to reagents, materials, and analysis tools. RJ wrote the manuscript. HK co-wrote the manuscript. All authors contributed to the article and approved the submitted version.

## Conflict of Interest

The authors declare that the research was conducted in the absence of any commercial or financial relationships that could be construed as a potential conflict of interest.

## Publisher’s Note

All claims expressed in this article are solely those of the authors and do not necessarily represent those of their affiliated organizations, or those of the publisher, the editors and the reviewers. Any product that may be evaluated in this article, or claim that may be made by its manufacturer, is not guaranteed or endorsed by the publisher.
